# A comparative genomics approach for identifying host-range determinants in *Streptococcus thermophilus* bacteriophages

**DOI:** 10.1038/s41598-019-44481-z

**Published:** 2019-05-29

**Authors:** Paula Szymczak, Martin Holm Rau, João M. Monteiro, Mariana G. Pinho, Sérgio Raposo Filipe, Finn Kvist Vogensen, Ahmad A. Zeidan, Thomas Janzen

**Affiliations:** 1 Bacterial Physiology, R&D, Chr. Hansen A/S, 2970 Hørsholm, Denmark; 2 Department of Food Science, University of Copenhagen, 1958 Frederiksberg, Denmark; 3 Laboratory of Bacterial Cell Biology, Instituto de Tecnologia Química e Biológica António Xavier, Universidade Nova de Lisboa, 2780-157 Oeiras, Portugal; 4 UCIBIO-REQUIMTE, Departamento de Ciências da Vida, Faculdade de Ciências e Tecnologia, Universidade Nova de Lisboa, 2829-516 Caparica, Portugal; 5 Laboratory of Bacterial Cell Surfaces and Pathogenesis, Instituto de Tecnologia Química e Biológica António Xavier, Universidade Nova de Lisboa, 2780-157 Oeiras, Portugal

**Keywords:** Super-resolution microscopy, Comparative genomics, Bacteriophages

## Abstract

Comparative genomics has proven useful in exploring the biodiversity of phages and understanding phage-host interactions. This knowledge is particularly useful for phages infecting *Streptococcus thermophilus*, as they constitute a constant threat during dairy fermentations. Here, we explore the genetic diversity of *S. thermophilus* phages to identify genetic determinants with a signature for host specificity, which could be linked to the bacterial receptor genotype. A comparative genomic analysis was performed on 142 *S. thermophilus* phage genomes, 55 of which were sequenced in this study. Effectively, 94 phages were assigned to the group *cos* (DT1), 36 to the group *pac* (O1205), six to the group 5093, and six to the group 987. The core genome-based phylogeny of phages from the two dominating groups and their receptor binding protein (RBP) phylogeny corresponded to the phage host-range. A role of RBP in host recognition was confirmed by constructing a fluorescent derivative of the RBP of phage CHPC951, followed by studying the binding of the protein to the host strain. Furthermore, the RBP phylogeny of the *cos* group was found to correlate with the host genotype of the exocellular polysaccharide-encoding operon. These findings provide novel insights towards developing strategies to combat phage infections in dairies.

## Introduction

Bacteriophages represent a constant threat for the dairy industry worldwide. Infections of the bacterial starters with phages result in acidification failures, frequently leading to a lower quality of dairy products^1^. Phages infecting *Streptococcus thermophilus* are important due to the commercial use of thermophilic starter cultures for the production of yoghurt and various types of cheese^2–4^.

Advances in genome sequencing technologies and bioinformatic tools enable in-depth exploration of dairy phage biodiversity. Genomic studies provide insights into the evolution and relatedness of phages, rendering fast and precise phage taxonomic schemes. These studies are also useful to elucidate mechanisms of phage-host interactions, and this knowledge is essential for the rational design of novel anti-phage strategies^1,5^. Such efforts include designing PCR methods for phage monitoring^6–9^, tracking the dynamics of the phage community during dairy fermentations^10^, identifying groups of genes with host-specificity signatures^11^, or optimizing starter rotation schemes by selecting phage-unrelated strains^12,13^.

Genomic studies require access to comprehensive genomics data. As of October 2018, the GenBank database comprised 87 publicly available *S. thermophilus* phage genomes^14–31^. Phages infecting *S. thermophilus* belong to the *Siphoviridae* family of the *Caudovirales* order^32^ and are currently differentiated into four groups: the two dominating groups termed *cos* and *pac*^33^, as well as the 5093 group^18,30^, and the 987 group^17,18^. Each group of dairy streptococcal phages displays individual characteristics, which is reflected in their genetic diversity and morphological features, including host-recognition features on the tail-tip^9,17,18,30^.

Structures on the tail-tip, called antireceptors or receptor binding proteins (RBP), are known to mediate host recognition^34–36^. Characterization of an RBP gene in *cos*-group phages revealed three characteristic regions: (i) the conserved region, which corresponds to the amino-terminus of the protein, (ii) the first variable region (VR1), which is only present in some phages, and (iii) the second variable region (VR2), which corresponds to the carboxy-terminus of the protein and was proposed to code for the host recognition domain^35^. The identity of the RBP gene in *pac*-group phages was predicted based on its genomic position and homology to the gene in *cos*-group phages^8,35^. RBP genes in the 987- and 5093-group phages were established by expressing and purifying phage proteins, followed by studying the inhibitory effect of these proteins on phage adsorption to the host strain^9,17^.

A phage RBP interacts with a specific component present on the bacterial cell surface. It was speculated that *cos* and *pac* phages adsorb to a carbohydrate receptor on the host cell surface^35,37,38^. In a recent study, we provide genetic and biochemical evidence that specific cell wall glycans, namely exocellular polysaccharides encoded by the *eps* operon and rhamnose-containing polysaccharides encoded by the *rgp* operon, can mediate phage adsorption to *S. thermophilus*^39^.

Understanding the affinity between a bacterial receptor and a phage RBP is essential for developing knowledge-based tools to counter the threat of phage infections in the dairy industry^1^. In *Lactococcus lactis*, a correlation between the bacterial cell wall polysaccharide genotype and the RBP phylogeny of phages from the 936 group was established^12^. A specific type of operon encoding the biosynthesis of a particular polysaccharide structure, the so-called pellicle, can be recognized by the specific phage RBP domain^12,13^. For *S. thermophilus*, the VR2 sequence of *cos*- and *pac*-group phages was used to correlate phages with their host-range. Although a relationship between sequence similarity and host specificity of phages was put forward, it cannot be extrapolated to all representatives of the two groups^3,8,9,35^.

An alternative host-range determinant could be established independent of RBP. Studies on *cos*- and *pac*-group phages suggested that two other genes, which code for the tape-measure protein (TMP) and the distal tail protein (Dit), could be related to the host specificity of these phages^40^. Similarly, the structural module, especially the region surrounding the major capsid protein- and the major tail protein-encoding genes, was observed to be a hotspot for genetic recombination among 936-group of *L. lactis* phages^13,41^. Structural proteins belong to a core genome in dairy lactococcal and streptococcal phages^9,13^. Those phage structures are believed to coevolve with the phage host and therefore, may play a role in phage-host interactions^13,41^.

The overall objective of this study is to investigate the genetic diversity of a *S. thermophilus* phage population to identify genetic determinants with a signature for host specificity, which could be linked to the receptor genotype in bacteria. Towards this goal, we expanded the database of *S. thermophilus* phage genomes by sequencing 55 new phages isolated from dairy fermentations that took place in different years and on different continents. By combining this dataset with publicly available genome sequences, a comparative genomic analysis of 142 phage genomes was performed. Subsequently, the role of a putative RBP of a *pac*-group phage in host recognition was verified by expressing and purifying a fluorescent derivative of RBP, followed by studying the protein adsorption to the host strain. Finally, the RBP phylogeny was linked to the genotype of the operon encoding biosynthesis of exocellular polysaccharides in *S. thermophilus*.

## Results

### General characteristics of the phages and their genomes

To investigate the dairy streptococcal phage population, the genomes of 55 *Streptococcus thermophilus* phages from the Chr. Hansen Phage Collection (CHPC) were sequenced in this study. The selected samples originated from cheese and yoghurt fermentations performed in various geographic locations, including Europe, North and South America, and they were isolated at various time-points, between 1995 and 2013 (Table 1). These features were expected to provide a broad perspective on genetic diversity and evolution of *S. thermophilus* phages.

**Table 1:** Characteristics of bacteriophage genomes from the Chr. Hansen Phage Collection sequenced in this study.

Phage	Host strain(s)	Group	Year of isolation	Country of origin	Product	Genome size (bp)	#ORFs	GC (%)	Accession #
CHPC1005	STCH_12	*cos*	2003	France	cheese	37,598	49	38	MH937483
CHPC1008	STCH_09 STCH_43	*pac*	2003	France	cheese	34,844	48	40	MH937484
CHPC1014	STCH_13	*cos*	2003	USA	cheese	35,260	49	38	MH937485
CHPC1027	STCH_12	*cos*	2004	Italy	cheese	35,928	48	38	MH937486
CHPC1029	STCH_13	*cos*	2004	Italy	cheese	35,920	47	39	MH937487
CHPC1033	STCH_12	*cos*	2004	Italy	cheese	36,827	49	38	MH937488
CHPC1034	STCH_13	*cos*	2004	Italy	cheese	33,826	42	39	MH937489
CHPC1036	STCH_18	*cos*	2004	Italy	cheese	36,333	50	38	MH937490
CHPC1037	STCH_34	*cos*	2005	USA	cheese	36,551	46	38	MH937491
CHPC1040	STCH_13	*cos*	2005	Italy	cheese	35,851	47	38	MH937492
CHPC1041	STCH_38	*cos*	2005	Italy	cheese	38,840	55	38	MH937493
CHPC1042	STCH_07	*pac*	2005	Italy	cheese	42,019	63	39	MH937494
CHPC1045	STCH_40	*cos*	2005	USA	cheese	34,096	46	38	MH937495
CHPC1046	STCH_14 STCH_39	*cos*	2005	USA	cheese	34,790	48	38	MH937496
CHPC1048	STCH_14 STCH_39	*cos*	2005	USA	cheese	34,812	48	38	MH937497
CHPC1057	STCH_09 STCH_43	*pac*	2005	France	yoghurt	34,845	47	40	MH937498
CHPC1062	STCH_31	*cos*	2005	France	cheese	40,037	56	38	MH937499
CHPC1067	STCH_12	*cos*	2005	France	cheese	34,355	45	39	MH937500
CHPC1073	STCH_12	*cos*	2006	France	cheese	34,017	47	38	MH937501
CHPC1083	STCH_12	*pac*	2006	USA	cheese	36,471	48	39	MH937502
CHPC1084	STCH_13	*pac*	2006	USA	cheese	36,776	49	39	MH937503
CHPC1091	STCH_04	*cos*	2006	France	yoghurt	37,028	49	38	MH937504
CHPC1109	STCH_36	*pac*	2006	USA	cheese	33,791	47	39	MH937505
CHPC1148	STCH_41	*cos*	2008	Germany	cheese	39,069	52	38	MH937506
CHPC1152	STCH_06	*pac*	2008	France	yoghurt	35,353	47	39	MH937507
CHPC1156	STCH_19	*cos*	2009	USA	cheese	34,912	48	38	MH937508
CHPC1230	STCH_45	*pac*	2012	USA	cheese	39,384	57	39	MH937509
CHPC1246	STCH_07	*pac*	2013	USA	cheese	36,876	55	39	MH937510
CHPC1247	STCH_07	*pac*	2013	France	cheese	35,543	50	39	MH937511
CHPC1248	STCH_07	*pac*	2013	France	cheese	38,383	56	39	MH937457
CHPC572	STCH_24	*cos*	N/A	Italy	cheese	37,005	51	38	MH937458
CHPC595	STCH_25	*cos*	N/A	Argentina	cheese	35,697	49	39	MH937459
CHPC640	STCH_26 STCH_28	*pac*	N/A	Italy	cheese	40,404	57	38	MH937460
CHPC642	STCH_17	*cos*	N/A	Italy	cheese	35,715	52	38	MH937461
CHPC663	STCH_29	*cos*	1995	Italy	cheese	38,531	54	38	MH937462
CHPC676	STCH_26 STCH_28	*pac*	N/A	Italy	cheese	40,402	55	38	MH937463
CHPC869	STCH_44	*pac*	1998	France	yoghurt	37,080	52	39	MH937464
CHPC873	STCH_42	*cos*	1998	France	cheese	38,258	53	38	MH937465
CHPC875	STCH_23	*cos*	1998	France	cheese	36,576	51	39	MH937466
CHPC877	STCH_32 STCH_33	*cos*	1998	France	cheese	39,965	54	38	MH937467
CHPC879	STCH_20	*cos*	1998	France	cheese	36,011	48	38	MH937468
CHPC919	STCH_37	*cos*	2000	France	cheese	37,281	48	38	MH937469
CHPC925	STCH_21	*cos*	2000	France	cheese	34,759	47	38	MH937470
CHPC927	STCH_32 STCH_33	*cos*	2000	France	cheese	37,303	50	38	MH937471
CHPC928	STCH_22	*cos*	2000	France	cheese	34,022	43	38	MH937472
CHPC929	STCH_35	*pac*	2000	France	cheese	40,874	60	38	MH937473
CHPC930	STCH_30	*cos*	2000	France	cheese	36,350	49	38	MH937474
CHPC931	STCH_44	*pac*	2000	France	yoghurt	36,490	51	39	MH937475
CHPC933	STCH_02	*pac*	2001	France	yoghurt	32,182	40	40	MH937476
CHPC950	STCH_13	*cos*	2002	UK	cheese	35,299	52	39	MH937477
CHPC951	STCH_12	*pac*	2002	USA	cheese	36,471	49	39	MH937478
CHPC952	STCH_13	*pac*	2002	USA	cheese	36,775	49	39	MH937479
CHPC954	STCH_27	*cos*	2002	USA	cheese	37,464	47	38	MH937480
CHPC979	STCH_13	*cos*	2002	Germany	cheese	34,277	50	38	MH937481
CHPC982	STCH_13	*cos*	2002	France	cheese	35,246	48	38	MH937482

The investigated phages exhibited a narrow host-range. As verified in spot tests with 37 industrial *S. thermophilus* strains, the studied phages infected their primary host and, in a few cases, only one additional strain (Table S1). Three of the tested strains, STCH_07, STCH_12, and STCH_13, were susceptible to infection by four, seven, and nine specific phages, respectively. These phages were included in the study to examine the genotypic similarity of phages that infect the same host.

The overall genome architecture of the sequenced phages was comparable to the *S. thermophilus* phage genomes currently available in GenBank. The genome length varied from 32 to 42 kb (average 36.5 kb) and phage genome sequences had a GC content of approximately 38%. Forty to 63 (average 50) coding sequences (CDS) were identified in each genome using RASTtk^42^. In a previous study, the selected phages were subjected to *pac*- and *cos*-grouping using the published multiplex PCR method^18^. The results of the assay classified 36 phages into the *cos* group and 19 phages into the *pac* group. The detailed information on the phages sequenced in this study is presented in Table 1.

### Grouping of the *S. thermophilus* phage population

**Figure 1:**
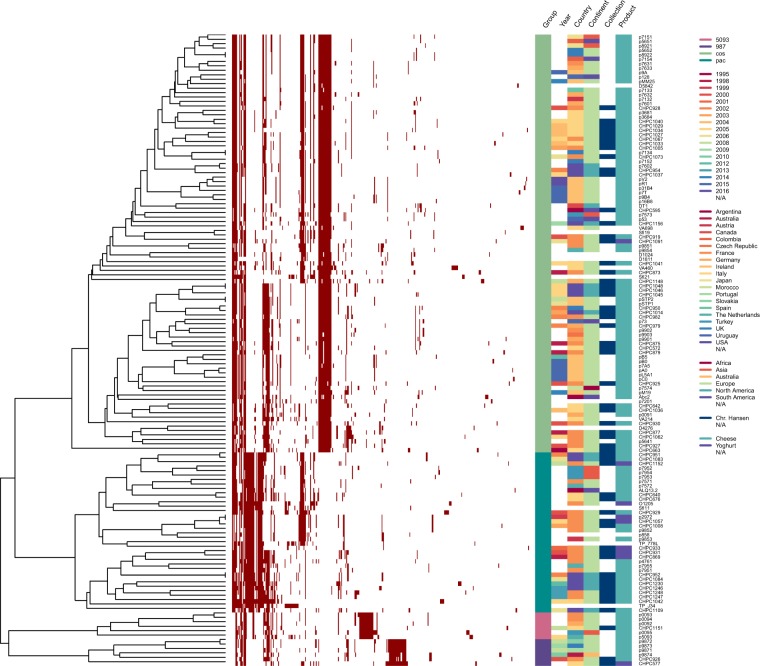
Hierarchical clustering of a gene content variation among 142 *S. thermophilus* phages. Pangenome information on the presence or absence of a representative gene of an orthologous group was employed for functional clustering of phages. Gene presence or absence is denoted in the heatmap by red and white colour, respectively. Metadata of the following characteristics is integrated in the dendrogram: the group of a phage (Group), the year and the geographic location of sample isolation (Year, Country, Continent), the type of dairy fermentation the phages were isolated from (Product), the industrial collection of phages (Collection). The varieties within each feature are marked with different colours.

A comparative genomic analysis was performed with 142 *S. thermophilus* phage genomes: 55 phage sequences obtained in this study and 87 phage genomes available in GenBank (Table S2). This analysis aimed primarily at establishing the genetic relatedness of phages, which should provide a more comprehensive and accurate grouping. The pangenome covering all 142 phages was created through the identification of orthologous gene groups based on sequence similarity (>50% identity) and sequence coverage (>50%). The resulting pangenome information on the absence or presence of orthologous gene groups within the genomes allowed the hierarchical clustering of phages based on their gene content (Fig. 1).

Based on pangenome clustering, *S. thermophilus* phages were divided into four main clusters, which corresponded to the previously proposed groups: *cos*, *pac*, 5093, and 987 (Fig. 1). In addition, two sub-clusters of the *cos* lineage and three sub-clusters of the *pac* lineage were observed, which indicated a potential for further division within the two dominating groups. Notably, only a limited number of gene products were complementary within each of the four major clusters, highlighting the mosaic nature of phage genomes.

### Factors that shape the genetic relatedness of phages

To identify possible factors that influence the genetic makeup of phages, the visualization of the functional clustering was amended by metadata with the following information: (i) the type of fermentation processes the phages were isolated from, (ii) the industrial collection that phages belong to, (iii) the geographic location, and (iv) the year of sample isolation (Fig. 1). Several relatedness patterns were observed based on the generated network.

Phages with common characteristics, *e.g*. isolated in the same year or from the same continent, were scattered across the dendrogram. However, samples that originated from one geographic location, but were isolated at various time-points, clustered closely together (Fig. 1, features ‘Year’ and ‘Country’). This observation supports the hypothesis that phage particles with nearly unchanged genotype persist in the processing environment for extended periods of time^43,44^.

Nine phages from the analysed population were isolated from yoghurt fermentations performed with strains that possess texturizing properties, while the rest of the isolates originated from whey samples of cheese fermentations. Seven of the nine yoghurt-originated phages belonged to the *pac* group (Fig. 1, feature ‘Product’), which may suggest that *pac*-group phages have an advantage over *cos*-group phages for infecting free exopolysaccharide-producing strains.

Phages from CHPC clustered closely together, irrespectively of their geographic origin (Fig. 1, features ‘Continent’ and ‘Collection’). These phages were isolated from the industrial fermentations performed with starter cultures provided by Chr. Hansen. The same bacterial cultures were used in different locations worldwide, which could explain the dissemination of closely related phages across the globe. Thus, the industrialization of the dairy production could be a factor that may shape the diversity of the *S. thermophilus* phage population.

### Core genome as a host-range determinant

To further investigate the genetic similarity of *S. thermophilus* phages, the core genome was determined from the constructed pangenome of the 142 phage genome sequences. Based on the results, no conserved genes were identified across all members of the examined population. However, one gene coding for a phage protein was present in all members except for phage D1811. This gene had distal genomic location and a representative is ORF43 of CHPC1040. In total 361 genes were covered by the pangenome, and of these 30% were unique genes. These results revealed a significant genetic heterogeneity of *S. thermophilus* phages.

Even though core genes covering all phage groups were not identified, core genes within each group were recognized. Phages of the two dominating groups *cos* and *pac* had 13 core genes each. Phages of the groups 987 and 5093 had 24 and 28 core genes, respectively, which is likely a reflection of the fewer genomes within these groups or that these groups are more genetically related. The genetic elements of the identified core genomes primarily corresponded to the genes coding for structural proteins and genome packaging. Since the *cos*- and *pac*-group phages are the most frequently isolated dairy streptococcal phages, we opted to analyse those two groups in detail.

We questioned whether the similarity within the *cos* and *pac* core genomes can be attributed to the host-range of the phages. This information could be used to establish starter rotation schemes in dairy plants that could prevent acidification failures due to phage infection. Therefore, phage phylogeny was constructed based on the 13 core genes of the 94 *cos*-group phages and the 13 core genes of the 36 *pac*-group phages (Figure S1).

The analyses showed that phages that infected the same host(s) clustered together on well supported branches. Within the *cos* group, we identified 16 instances of a shared host, *i.e*. cases where two or more phages infected a single strain, or a pair of phages infected two strains. In 13 of these cases, phages that infected the same strain were phylogenetically related (Figure S1a). Similarly, *pac*-group phages that infected the same strain were located on the same branches, with only a single exception observed (Figure S1b).

### Identifying the antireceptor gene

**Figure 2:**
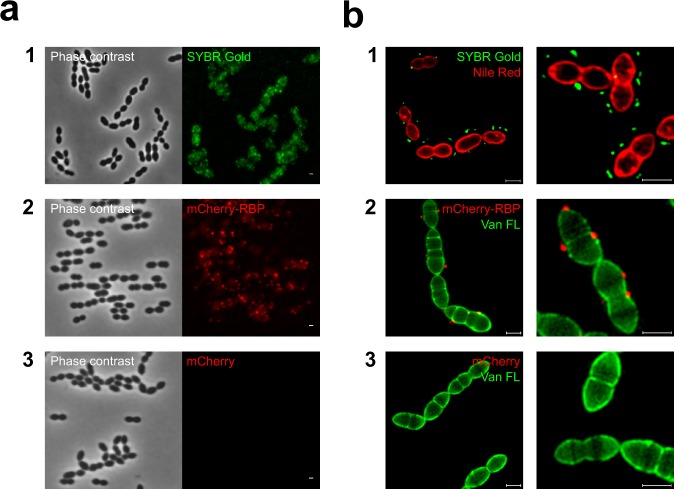
Fluorescence imagining of a phage and a phage antireceptor (RBP) binding to *S. thermophilus*. Strain STCH_12, phage CHPC951, a fluorescence derivative of the RBP of phage CHPC951, and a purified mCherry protein were used in the experiments. (**a**) Imagining under a conventional fluorescence microscope: (1) adsorption of SYBR Gold DNA-labelled phages (green) to the host strain; (2) adsorption of mCherry-tagged RBP (red) to the bacterial cells; (3) no adsorption of mCherry proteins to the bacterial cells. (**b**) Super-resolution structured illumination microscopy (SIM) imagining: (1) bacterial cells were labelled with Nile Red (red) and mixed with SYBR Gold-DNA labelled phages (green); (2) bacterial cells were labelled with Van FL (green) and mixed with mCherry-tagged RBP (red); (3) bacterial cells were labelled with Van FL (green) and mixed with mCherry proteins (red). Scale bars: 1 µm.

RBP is the factor that mediates host recognition. Thus, a host-related grouping of the *cos*- and *pac*-group phages was expected to be generated by comparing the RBP gene sequence. The identity of the RBP in *cos*-group phages was established previously^35^. Here, we focused on confirming the function of a putative RBP gene in the *pac*-group phages, which was predicted based on the genomic position and the similarity to the *cos*-group phages. Moreover, we wanted to specify whether a single protein encoded by the RBP gene is sufficient to create the irreversible attachment to the phage receptor on the bacterial cell surface. To that end, we constructed and purified a fluorescent variant of a putative RBP from *pac*-group phage CHPC951 (ORF20) as described in Materials and Methods. Subsequently, we visualized the adsorption of phage CHPC951 and its recombinantly produced RBP to *S. thermophilus* host strain STCH_12. The images were acquired using both conventional fluorescence microscopy and super-resolution structured illumination microscopy (SIM) (Fig. 2).

Interactions between the phage and its host strain, as well as between the mCherry-tagged RBP and the bacterial cells were observed in the microscopy assays. A fluorescent signal was detected around the bacterial cells when strain STCH_12 was mixed with SYBR Gold DNA-labelled phage particles (Fig. 2a, panel 1) and with the mCherry-tagged derivative of the phage RBP (Fig. 2a, panel 2). As visualized by SIM, phage particles and mCherry-tagged RBP bound to the host cells (Fig. 2b, panels 1 and 2). For the intact phages, the green fluorescent signal originating from a phage capsid, which contained DNA labelled with SYBR Gold, was localized 0.21 ± 0.07 µm (average ± SD, n = 80 phage capsids) from the bacterial cell surface (Fig. 2b, panel 1). This distance is in accordance to the values determined by electron microscopy for the length of the phage tail^39^. The red fluorescent signal of mCherry-tagged RBP was localized directly on the cell surface (Fig. 2b, panel 2). The purified mCherry protein, used as a negative control for the assays, did not bind to the bacterial cells (Fig. 2a,b, panel 3). These results highly suggested that the putative RBP gene in the *pac*-group phage encodes the receptor binding protein and that it is sufficient to attach the mCherry-tagged protein (or a phage) on the bacterial cell surface.

### Antireceptor phylogeny correlates with host specificity

**Figure 3:**
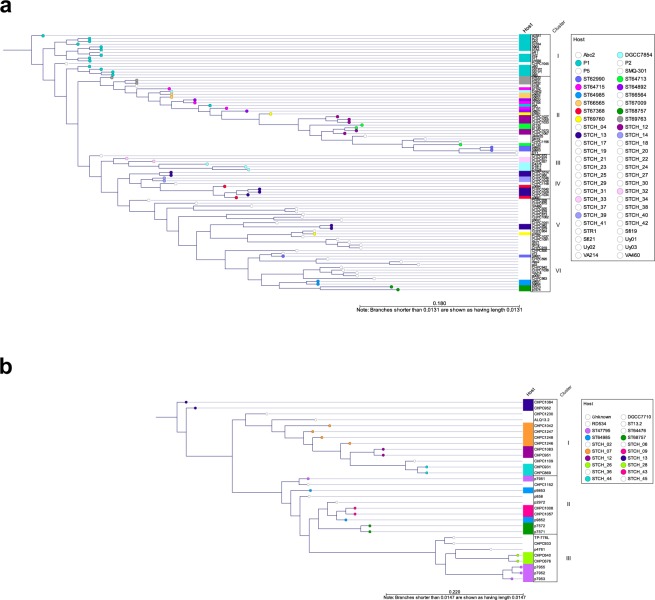
Phylogenetic comparison of the antireceptor (RBP) from *cos*- and *pac*-group *S. thermophilus* phages. The phylogenetic tree was inferred from alignment of the RBP protein sequence. (**a**) The RBP phylogeny of *cos*-group phages. A colour-coding is used to highlight a host strain that is infected by two or more phages (STCH_12, STCH_13, ST66565, ST67368, ST62990, ST68757, ST64713, ST69760, ST64715, ST69763, ST64892, ST64985, DGCC7854, P1), as well as pairs of strains that are infected by two different phages (STCH_32 and STCH_33, STCH_14 and STCH_39). RBP phylogeny clusters (I to VI) are assigned. (**b**) The RBP phylogeny of *pac*-group phages. A colour-coding is used to highlight a host strain that is infected by two or more phages (STCH_07, STCH_12, STCH_13, STCH_44, ST649885, ST47795, ST68757), as well as pairs of strains that are infected by two different phages (STCH_26 and STCH_28, STCH_09 and STCH_43). RBP phylogeny clusters (I to III) are assigned.

To verify the hypothesis that the RBP can be a gene with a significant signature of host specificity, a phylogenetic analysis of the RBP of the *cos*- and *pac*-group phages was performed (Fig. 3, ORFs used in the analysis are listed Table S2). Two *cos*-group phages (7201, VA698) and five *pac*-group phages (O1205, Sfi11, CHPC929, 7954, and TP-J34) were excluded from the analysis because the sequence similarity of their RBP was below 30% in comparison to the other RBPs used in the study. Thus, homology with other RBPs, which is a prerequisite for a phylogenetic analysis, could not be established. In 12 out of 16 cases of shared host-ranges within the *cos* group, phages that infected the same strain(s) clustered together, based on the RBP phylogeny (Fig. 3a). For the *pac* group, the host specificity correlated well with the RBP phylogeny, with only one exception (Fig. 3b). The correlation between the RBP phylogeny and the host-range of the phages was comparable to the correlations obtained from the core genome analyses.

### Correlation of the antireceptor phylogeny and the *eps* and *rgp* operon genotype

Our next goal was to identify the genetic determinants of phages that would correlate with the receptor genotype in bacteria. Therefore, we investigated whether there is a link between the phage RBP phylogeny and the genotype of the *eps* and *rgp* operons, presumably encoding phage receptors of *S. thermophilus*. To address this query, 43 phage genomes from CHPC, 27 *cos*- and 16 *pac*-group phages, were selected from the original dataset, and analysed together with their 23 host strains, the genomes of which are part of the internal Chr. Hansen Collection.

**Figure 4:**
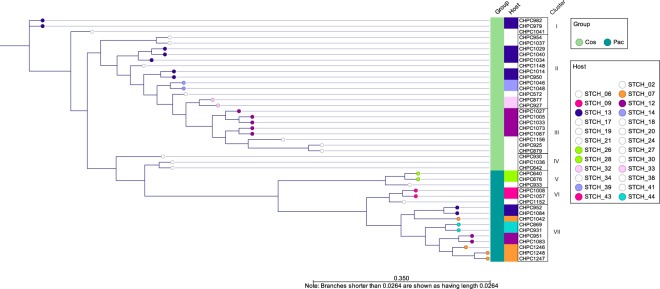
Phylogenetic comparison of the antireceptor (RBP) from the selected *cos-* and *pac*-group *S. thermophilus* phages. The phylogenetic tree was inferred from alignment of the RBP protein sequence. A colour-coding is used to highlight a host strain that is infected by two or more phages, as well as a pair of strains that are infected by a pair of phages. Metadata on the phage group (*cos* or *pac*) is integrated in the dendrogram. RBP phylogeny clusters (I to VII) are assigned.

Seven homology clusters were identified based on the RBP phylogeny of the selected phages (Fig. 4). Phages from the *cos* and *pac* groups were separated into different clusters. The *cos*-group phages used in the analysis belonged to the RBP lineages I to IV, while the *pac*-group phages belonged to the RBP lineages V to VII. Phages that infected the same strain(s) belonged to the same RBP lineage, apart from phages that infected strains STCH_12 and STCH_13. The *pac*-group phages of these two strains belonged to the RBP lineage VII. The *cos*-group phages of STCH_12 belonged to the RBP lineage III, while the *cos*-group phages of STCH_13 belonged to the RBP lineages I and II. The analysis confirmed that RBP phylogeny correlates well with the host association. However, the observed division between *cos*- and *pac*-group phages indicated that RBPs of these two phage groups significantly differ, which can result in recognizing different cellular components.

**Figure 5:**
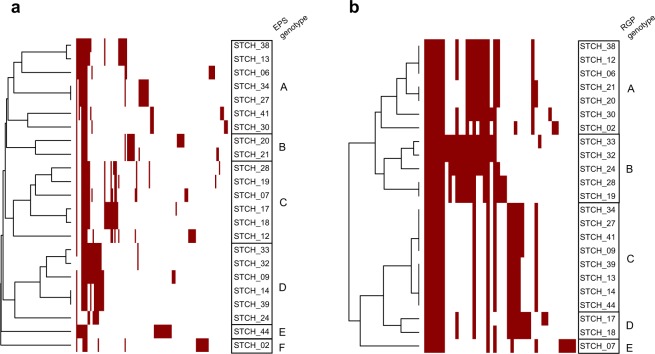
Hierarchical clustering of a gene content variation among *eps* and *rgp* gene clusters of 24 *S. thermophilus* strains. Gene presence or absence is denoted in the heatmap by red and white colour, respectively. (**a**) EPS genotypes (A to F) and (**b**) RGP genotypes (A to E) are assigned.

The *eps* and *rgp* gene cluster contents of the 23 *S. thermophilus* strains, which are hosts for the selected phages, were identified as specified in Materials and Methods. The *eps* clusters contained 17.7 genes on average, of which 13 genes were the minimal number of genes identified, while the *rgp* clusters contained 16.8 genes on average, with a lowest number of 14. In certain strains, the *eps* or *rgp* operon was located in two contigs and thus, genes could be missing in these gene clusters. Hierarchical clustering of the strains based on the presence or absence of orthologous protein groups encoded by the *eps* and *rgp* gene clusters was performed (Fig. 5).

Six clusters of the *eps* operon and five clusters of the *rgp* operon were identified (Fig. 5). The genotypes were designated as EPS types A to F (for exopolysaccharides biosynthesized via the *eps* operon) and RGP types A to E (for rhamnose-containing polysaccharides biosynthesized via the *rgp* operon). Strains that possessed one EPS genotype, possessed separate RGP genotypes (Table 2). An additional hierarchical clustering based on the bacterial pangenome showed that core genome similarity does not reflect the *rgp* or *eps* gene content similarity (Figure S2). This observation is in line with the notion that genes of the *rgp* and *eps* operons are often acquired by horizonal gene transfer. Therefore, homologs with similar functions can be phylogenetically more distant than other genes within the genome. The established relationships based on the *eps* and *rgp* gene cluster contents could reflect the biosynthesis of similar polysaccharide structures by strains that belong to the same EPS or RGP genotype.

**Table 2:** List of *S. thermophilus* phages with the corresponding RBP phylogeny cluster (I to VII), as assigned in Fig. 4, and their host strains with the corresponding EPS genotype (A to F) and RGP genotype (A to E), as assigned in Fig. 5.

RBP phylogeny cluster	Phage	Host strain	EPS genotype	RGP genotype
I^a^	CHPC979	STCH_13	A	C
	CHPC982			
	CHPC1041	STCH_38	A	A
II^a^	CHPC954	STCH_27	A	C
	CHPC1037	STCH_34	A	C
	CHPC572	STCH_24	D	B
	CHPC877	STCH_32	D	B
	CHPC927	STCH_33	D	B
	CHPC1014	STCH_13	A	C
	CHPC1029			
	CHPC1034			
	CHPC1040			
	CHPC950			
	CHPC1148	STCH_41	A	C
	CHPC1046	STCH_39	D	C
	CHPC1048	STCH_14	D	C
III^b^	CHPC1005	STCH_12	C	A
	CHPC1027			
	CHPC1033			
	CHPC1067			
	CHPC1073			
	CHPC1156	STCH_19	C	B
	CHPC879	STCH_20	B	A
	CHPC925	STCH_21	B	A
IV^b^	CHPC930	STCH_30	A	A
	CHPC642	STCH_17	C	D
	CHPC1036	STCH_18	C	D
V	CHPC933	STCH_02	F	A
	CHPC640	STCH_28	C	B
	CHPC676			
VI	CHPC1152	STCH_06	A	A
	CHPC1008	STCH_09	D	C
	CHPC1057			
VII	CHPC1083	STCH_12	C	A
	CHPC951			
	CHPC1084	STCH_13	A	C
	CHPC952			
	CHPC869	STCH_44	E	C
	CHPC931			
	CHPC1042	STCH_07	C	E
	CHPC1246			
	CHPC1247			
	CHPC1248			

^a^Probability of only A and D *eps* gene clusters for a group size of RBP cluster I and II: < 3E-7. ^b^Probability of only B and C *eps* gene clusters, except one, for a group size of RBP cluster III and IV: < 2E-4.

Certain correlations between the genotype of the *eps* operon and the RBP phylogeny of *cos*-group phages were observed (Table 2). Phages of RBP phylogeny cluster I and II infect strains that possess EPS type A and D, while phages of RBP lineages III and IV preferentially infect strains of EPS type B and C.

## Discussion

In this work, the use of comparative genomics enabled accurate grouping of 142 *S. thermophilus* phages and guided the identification of genetic determinants with a signature of host specificity. Four groups of *S. thermophilus* phages were defined, and additional subgroups were observed within the two dominating groups, known as *cos* and *pac*. The core genome phylogeny and the RBP phylogeny of phages from these two groups could be correlated with the host-range of phages. The role of RBP of *pac*-group phage CHPC951 in creating the irreversible binding to the host cell surface receptor was confirmed. Finally, a correlation between the *eps* operon genotype and the RPB phylogeny of phages from the *cos* group was observed.

The comparative analysis of *S. thermophilus* phage genomes confirmed that this population can be divided into the previously defined groups *cos*, *pac*, 5093, and 987^9,18^. Considering the growing number of phages of the groups 987 and 5093, which also use *pac* and *cos* DNA packaging mechanisms, the conventional classification of *S. thermophilus* phages based on DNA packaging mechanisms (*cos* and *pac*) and structural protein composition should be revised^33^. Therefore, we propose new names for the two dominating groups: the *pac* group to be described as group O1205, because phage O1205 was the first *pac*-group representative defined^14^, and the *cos* group to be described as group DT1, because phage DT1 was used as a model of *cos*-group phages in several studies^24,28,35,40^. The novel nomenclature will be more accurate in reflecting the current grouping of *S. thermophilus* phages and allow for further subgrouping within the four major groups.

The results of this study unveiled the mosaic nature of phage genomes and the conservation across the structural genes within the four defined groups, which is in accordance with a previous report^9^. The relatedness of *S. thermophilus* phages can be predominantly shaped by industrialization. This observation is not surprising, since the investigated phages originate from industrial fermentations. Indeed, multilocus sequence typing of *S. thermophilus* strains showed that industrial cultures cluster independently from their geographic origin and fermented product^45^. The dissemination of phages in industrial fermentations can be due to *e.g*. the addition of whey protein concentrates to cheese milk. These milk by-products were shown to be a rich source of phages^46,47^. Thus, global movements of dairy starter cultures and use of products derived from whey likely lead to exchanging phage genomic information and distributing genetically similar phages in different locations across the globe^13^.

The phylogenetic analyses based on the core genome or the RBP were equally effective in representing the host specificity of phages. Hence, they could both possibly serve to generate predictions on potential phage-bacteria interactions. In this study, we aimed at establishing the relatedness of phages that would correlate with the type of the phage receptor on the bacterial cell surface. The RBP sequence was proven to encode the protein that efficiently adsorbs to a component on the bacterial cell surface. Although some other gene products could additionally assist in host recognition^40^, the single RBP was sufficient to create the irreversible interaction with the bacterial cell surface receptor. Therefore, the RBP phylogeny was used to link phage relatedness with the receptor genotype of bacteria.

The results of this study indicated the possibility of the unique relationships between the *cos*-group phage RBP structure and the exocellular polysaccharide structure biosynthesized by the *eps* operon in *S. thermophilus*. Indeed, putative receptor mutants of *S. thermophilus* were shown to acquire mutations in genes belonging the *eps* operon as a response for the infection by *cos*-group phages^39^. The RBP phylogeny of the *pac*-group phages could not be correlated with specific genotypes of the *eps* and *rgp* operons. However, in this study, a limited pool of 16 *pac*-group phages was used to investigate the association with the receptor genotype. Comparing larger dataset of phage and host genomes would possibly result in a more accurate clustering based on the RBP phylogeny of *pac*-group phages, and lead to revealing association with the specific RGP or EPS genotypes.

Notably, the clustering based on the RBP phylogeny of the selected phages from the *cos* and *pac* groups suggested that phages belonging to different groups recognize diverse receptors on the host cell surface. A single strain likely possesses two types of phage receptors, as a putative receptor mutant of *S. thermophilus* was reported to acquire resistance towards *cos*-group phages but remained sensitive towards *pac*-groups phages^48^. If the receptors of *cos*-group phages are polysaccharides biosynthesized via the *eps* operon, the *pac*-group phages could recognize RGP biosynthesized via the *rgp* operon.

In this study, it was assumed that a specific *eps* genotype is responsible for the biosynthesis of a particular polysaccharide structure. To verify this notion and elucidate genotype-phenotype associations in general, further studies on glycobiology in *S. thermophilus* are required. The host specificity of phages, which was used for correlating RBP phylogeny with the EPS and RGP genotype, was established based on the spot test, *i.e*. the ability of phages to form plaques with a bacterial strain. However, successful phage infection is dependent on the receptor recognition as well as additional factors, such as presence of intracellular phage-resistance mechanisms^49^. Thus, phages with close RBP phylogeny, which did not form plaques with each other’s hosts, could still recognize a specific polysaccharide encoded by a similar *eps* or *rgp* operon. To confirm this hypothesis, further studies should be undertaken to examine the adsorption of phages from a given RBP cluster to the strains with the associated EPS genotype.

In conclusion, the data generated in this study could be successfully used for accurately grouping *S. thermophilus* phages and correlating the *cos*-group phage RBP phylogeny with the genotype of the *eps* operon. These results should aid in improving starter rotation schemes as well as the selection of strains for culture development.

## Materials and Methods

### Bacteria, phages, and growth conditions

*Streptococcus thermophilus* strains and phages used for this study are listed in Table 1. Strains were stored at −40 °C in growth medium supplemented with 15% (wt/vol) glycerol and cultured overnight at 37 °C in LM17 broth (M17 broth [Oxoid, Denmark] with 2% [wt/vol] lactose) or anaerobically at 37 °C on LM17 agar plates (M17 agar [Oxoid] with 2% [wt/vol] lactose). For experiments with phages, the growth medium was additionally supplemented with 10 mM CaCl_2_ and 10 mM MgCl_2_ (LM17-Ca/Mg). Phages were propagated on their corresponding host as previously described^18^ and stored at 4 °C.

Phage titers as well as the host ranges of investigated phages with bacterial strains were determined by using the double agar overlay spot test, as described before^50^. Following overnight incubation under the appropriate growth conditions, the plaque forming units (PFU) per milliliter were calculated.

Competent cells of *Escherichia coli* and plasmids used for the cloning procedure were stored at −80 °C. Transformants were selected on LB-Amp agar plates (LB agar [Difco, USA] with 100 µg/ml Ampicillin) and grown in LB-Amp broth (LB broth [Difco] with 100 µg/ml Ampicillin) at 37 °C with aeration at 150 rpm.

### Phage genome sequencing

DNA was isolated from the 55 phages listed in Table 1 and whole genome sequencing was performed using the Illumina MiSeq platform with 2 × 250 bp paired end sequencing (Illumina, USA), as described previously^18^. Sequencing data were processed using CLC Genomics Workbench 8.5 (Invitrogen, Denmark), as described before^18^. The genome fragments with low coverage (threshold 100 reads) were additionally verified by Sanger sequencing (Macrogen, The Netherlands).

### Genomic analysis

Open reading frames (ORFs) in all genomes, including the publicly available ones, were identified and functionally annotated through the RASTtk pipeline^42^, with default parameters. The translated protein sequences of protein-encoding genes were employed for pangenome construction. Orthologs were identified by blast-based bidirectional best hit (BBH) using Proteinortho^51^ with 50% identity, 50% coverage and 1.0 similarity cut-offs. The obtained pangenome was visualized by hierarchical clustering with the Jaccard distance metric and UPGMA linkage method, using the hclust and heatmap.2 functions in R.

As no core genes could be identified in the pangenome of the four phage groups, the core genes within each phage group were used to construct phage-group specific phylogeny. For that, the nucleotide sequences of concatenated core genes were first aligned using prank^52^, with the -F option and otherwise default parameters. Phylogeny was then inferred by the Maximum-Likelihood method using RAxML-NG (https://github.com/amkozlov/raxml-ng), which is based on RAxML^53^, with the GTRGAMMA nucleotide substitution model and default parameters. For constructing the phylogeny of the RBP protein within the phage genomes, translated protein sequences of the corresponding genes were aligned with prank and phylogeny was inferred using RAxML-NG as described above, but with the WAG substitution model instead of the GTR model. This was performed for the *pac* and *cos* phage groups together or individually. Phylogeny clusters were determined by comparing the same phylogenetic tree in phylogram and radial layouts.

The hierarchical clustering of the *eps* and *rgp* gene cluster content of selected phage host strains was initiated by identifying the location of the *eps* and *rgp* cluster genes within the bacterial genomes. This was performed through a blast search for two flanking genes, *i.e. epsA* and predicted membrane protein (TMS6) for *eps* clusters, and *radC* and bactoprenol glucosyl transferase for *rgp* clusters, as annotated by RAST^54^. Ortholog groups of the proteins encoded by the genes within the *eps* and *rgp* clusters were determined by blast-based BBH with 40% amino acid sequence identity, 80% coverage and 1.0 similarity cut-offs. A lower percent identity was employed here as the genes within these clusters are often acquired by horizontal gene transfer. Therefore, homologs with similar function are potentially phylogenetically more distant than other genes within the genome. The pangenome of the selected bacterial strains was determined with the same methods and parameters, as described above for the phage pangenome. Subsequent hierarchical clustering of the bacterial pangenome, *eps* and *rgp* gene clusters were performed as described for the phage pangenome. Probabilities of selected RBP vs. *eps* or *rgp* cluster group distributions were calculated as sampling without replacement.

### Fluorescent derivative of a phage antireceptor

A fluorescent derivative of RBP of *pac*-group phage CHPC951 was cloned and expressed using a commercially available vector pET21a (Novagen, Germany) that was transformed into competent cells of *E. coli*. The version of mCherry fluorescent protein used for this work was reported before^55^. PCR amplifications were carried out using Phusion High-Fidelity DNA Polymerase (Thermo Fisher Scientific, USA). Restriction and ligation enzymes (New England Biolab, USA) were used according to the manufacturer’s protocol. PCR amplicons were purified using Wizard SV Gel and PCR Clean-up System (Promega, USA). Plasmid DNA was isolated using Wizard Plus SV Minipreps (Promega). Sequences of primers used for the cloning procedure are listed in Table 3.

**Table 3:** List of primers used in this study.

Primer name	Sequence, 5′–3′	Reference
mCherry_FWD	GCGGATCCGTGAGCAAGGGCGAGGAGGA TAACATGG	This study
mCherry-His_REV	CGCGGCCGCAAGCTTTTAGTGATGGTGAT GGTGATGCTTGTACAGCTCGTCC	
mCherry_REV	GTGAATTCCCTTCCCTCGATCCCGAGATTG TTGTTCTTGTACAGC	
RBP951_FWD	GGGATCGAGGGAAGGGAATTCACCTTGCT AACAATTCACGACG	
RBP951_REV	CGCGGCCGCAAGCTTTTAGTGATGGTGATG GTGATGTGCACCTCCTACATATCTTATGACG	
T7 promoter_FWD	TAATACGACTCACTATAGG	Novagen
T7 terminator_REV	GCTAGTTATTGCTCAGCGG	

For expression of mCherry not fused to any protein, mCherry sequence with a sequence encoding 6 × His C-terminal-specifying purification tag and an appropriate restriction recognition sequence was amplified using primers mCherry_FWD and mCherry-His_REV. The PCR amplicons were purified, restricted with appropriated enzymes, and ligated into vector pET21a.

For expression of mCherry fused to the phage RBP, mCherry sequence was amplified using primers mCherry_FWD and mCherry_REV. The RBP sequence of phage CHPC951 (ORF20) with a sequence encoding 6 × His C-terminal-specifying purification tag and an appropriate restriction recognition sequence was amplified from phage CHPC951 lysate using primers RBP951_FWD and RBP951_REV. The purified PCR amplicons were joined by an overlap PCR reaction using primers mCherry-FWD and RBP951_REV. The construct encoding mCherry fused to the N-terminal of phage RBP was purified, restricted with appropriate enzymes, and ligated into vector pET21a.

Plasmids were transformed and propagated in *E. coli* DH5α (Invitrogen). Ampicillin-resistant colonies were screened with primers T7 promoter_FWD and T7 terminator_REV. Constructs were confirmed by Sanger sequencing of the amplified fragments. Plasmids were isolated from positive colonies grown overnight in LB-Amp broth and transformed into competent cells *E. coli* BL21(DE3) (Novagen). Cells were grown in LB-Amp to OD_600_ of 0.5. Subsequently, the protein expression was induced by adding isopropyl β-D-1-thiogalactopyranoside (IPTG) to a final concentration 1 mM and incubating overnight under the appropriate growth conditions. Cells were harvested (7,000 rpm for 7 min at 4 °C) and washed twice with equilibration buffer (50 mM Na_2_PO_4_, 300 mM NaCl, pH 7.4), followed by lysing at French Press at 1000 psi. The lysate was centrifuged (16,000 rpm for 20 min at 4 °C) to remove cell debris and insoluble components of the sample.

The fluorescent proteins mCherry and mCherry-tagged derivate of the phage RBP were purified from the lysate using cobalt affinity resin (Talon; BD Biosciences, USA), due to the 6-His tag located at the C-terminal end of both proteins, eluted in equilibration buffer with the addition of 5 mM and 10 mM imidazole, and dialyzed overnight against phosphate-buffer saline (PBS) pH 6.0. Proteins were stored at 4 °C in PBS pH 6.0. The samples were separated on SDS-PAGE gels (Bio-Rad, USA) to confirm the presence of induced target proteins. Protein concentration was quantified using Nanodrop (Thermo Fisher Scientific).

### Fluorescence microscopy

To visualize adsorption of SYBR Gold DNA-labelled phages and the mCherry-tagged proteins to the bacterial cells, fluorescence microscopy experiments were performed. Bacterial cultures at exponential phase (OD_600_ = 0.5) were used for the assays. Prior to imaging, samples were immobilized on microscope slides covered with a thin layer of 1% agarose in PreC medium^56^.

Wide-field fluorescence microscopy was performed using Zeiss Axioplan 2 microscope equipped with a Plan-Neofluar objective (100×/1.3 oil Ph3) and a Zeiss Axiocam 503 mono camera (Zeiss, Germany) with 1 s exposure for SYBR Gold and 2 s exposure for mCherry. After acquisition, conventional fluorescence microscopy images were processed using ImageJ software^57^.

Super-resolution structured illumination microscopy (SIM) was performed in an Elyra PS.1 microscope (Zeiss) and visualized using 561-nm laser with 50 ms exposure for Nile Red and 488-nm laser with 50 ms exposure for SYBR Gold or 561-nm laser with 100 ms exposure for mCherry and 488-nm laser with 100 ms exposure for Van FL. Images were acquired using five grid rotations, followed by reconstruction and processing with ZEN software (black edition, version 14.0.0.201).

To visualize phage adsorption to the host strain, phage lysate was mixed 1000:1 with a 10-fold diluted SYBR Gold stock solution (Invitrogen) and incubated overnight in the dark at 4 °C^18,58^. For the wide-field fluorescence microscopy, bacterial cultures were mixed with SYBR Gold DNA-labelled phages at a multiplicity of infection approx. 10 (MOI, ratio of PFU to CFU). For SIM, bacterial cells were labelled with Nile Red (Invitrogen) at a final concentration of 2 µg/ml, for 5 min at room temperature with agitation in the dark, washed twice with LM17-CaMg broth, and mixed with SYBR Gold-labelled phages as specified above.

To visualize binding of the phage RBP to the host strain, the mCherry protein and the mCherry-tagged RBP of phage CHPC951 were used. For the wide-field fluorescence microscopy, the bacterial culture was centrifuged at 9,000 × g for 3 min. Cells were resuspended with mCherry and mCherry-tagged RBP at the final concentration 0.4 mg/ml, incubated for 5 min at room temperature and washed once with LM17-CaMg broth. For SIM, bacterial cultures were labelled with Van FL solution (1:1 mixture of vancomycin (Sigma, USA) and the fluorescent BODIPY FL conjugate of vancomycin (Molecular Probes, USA)) at a final concentration of 1 μg/ml, for 5 min at 37 °C with agitation^59^. Van FL-labelled bacteria were mixed mCherry and mCherry-tagged RBP as specified above.

### Accession numbers

Of the phage genomes sequenced in this study: MH937457 to MH937511. Accession numbers of the sequences of *eps* and *rgp* gene clusters compared in this study: MK483529 to MK483592.

## Supplementary information

Supplementary information

Tables S1, S2
