# Supplementary material for: A comparative genomics approach for identifying host-range determinants in Streptococcus thermophilus bacteriophages
Source: Sci Rep. 2019 May 29;9:7991. doi: 10.1038/s41598-019-44481-z (PMC6541646; doi:10.1038/s41598-019-44481-z)
Supplement: Supplementary file 1 — Supplementary information [file 41598_2019_44481_MOESM1_ESM.pdf]

Supplementary information

A comparative genomics approach for identifying host-range determinants in *Streptococcus thermophilus* bacteriophages

Paula Szymczak, Martin Holm Rau, João M. Monteiro, Mariana G. Pinho, Sérgio Raposo Filipe, Finn Kvist Vogensen, Ahmad A. Zeidan, Thomas Janzen

Tables in Excel file

Table S1. Host range of 55 *S. thermophilus* phages assessed against a panel of 37 *S. thermophilus* strains. The phages and strains belong to the Chr. Hansen Collection. Values in the table stand for phage titers calculated as PFU per ml. Color-coding was used to indicate positive phage infection: green - strains sensitive to the infection by two or more phages; yellow - strains sensitive to the infection by one phage. Blank areas indicate that strains were insensitive to the infection by a given phage.

Table S2. List of phages used in the genomic analyses in this study. ORFs used for the phylogenetic analysis of the receptor binding protein (RBP) were annotated through RASTtk pipeline (see Materials and Methods). Proteins excluded from the comparison due to the low similarity with other RBPs (threshold 30% similarity) are marked in gray.

Figure legends

Figure S1. Phylogram of the identified core genome of the *cos*- and *pac*-group *S. thermophilus* phages. (a) The phylogeny of the 94 *cos*-group phages were inferred from the nucleotide sequence of the 13 *cos*-group core genes. A colour-coding is used to highlight a host strain that is infected by two or more phages (STCH_12, STCH_13, ST66565, ST67368, ST62990, ST68757, ST64713, ST69760, ST64715, ST69763, ST64892, ST64985, DGCC7854, P1), as well as pairs of strains that are infected by two different phages (STCH_32 and STCH_33, STCH_14 and STCH_39). Core genome clusters (I to VI) are annotated. (b) The phylogeny of the 36 *pac*-group phages were inferred from the nucleotide sequence of the 13 *pac*-group core genes. A colour-coding is used to highlight a host strain that is infected by two or more phages (STCH_07, STCH_12, STCH_13, STCH_44, ST649885, ST47795, ST68757), as well as pairs of strains that are infected by two different phages (STCH_26 and STCH_28, STCH_09 and STCH_43). Core genome clusters (I to IV) are annotated.

Figure S2. Hierarchical clustering of a gene content variation among 24 *S. thermophilus* strains. Pangenome information on the presence or absence of a representative gene of an orthologous group was employed for functional clustering of strains. Gene presence or absence is denoted in the heatmap by red and white colour, respectively. Pangenome lineages (A to G) are assigned.

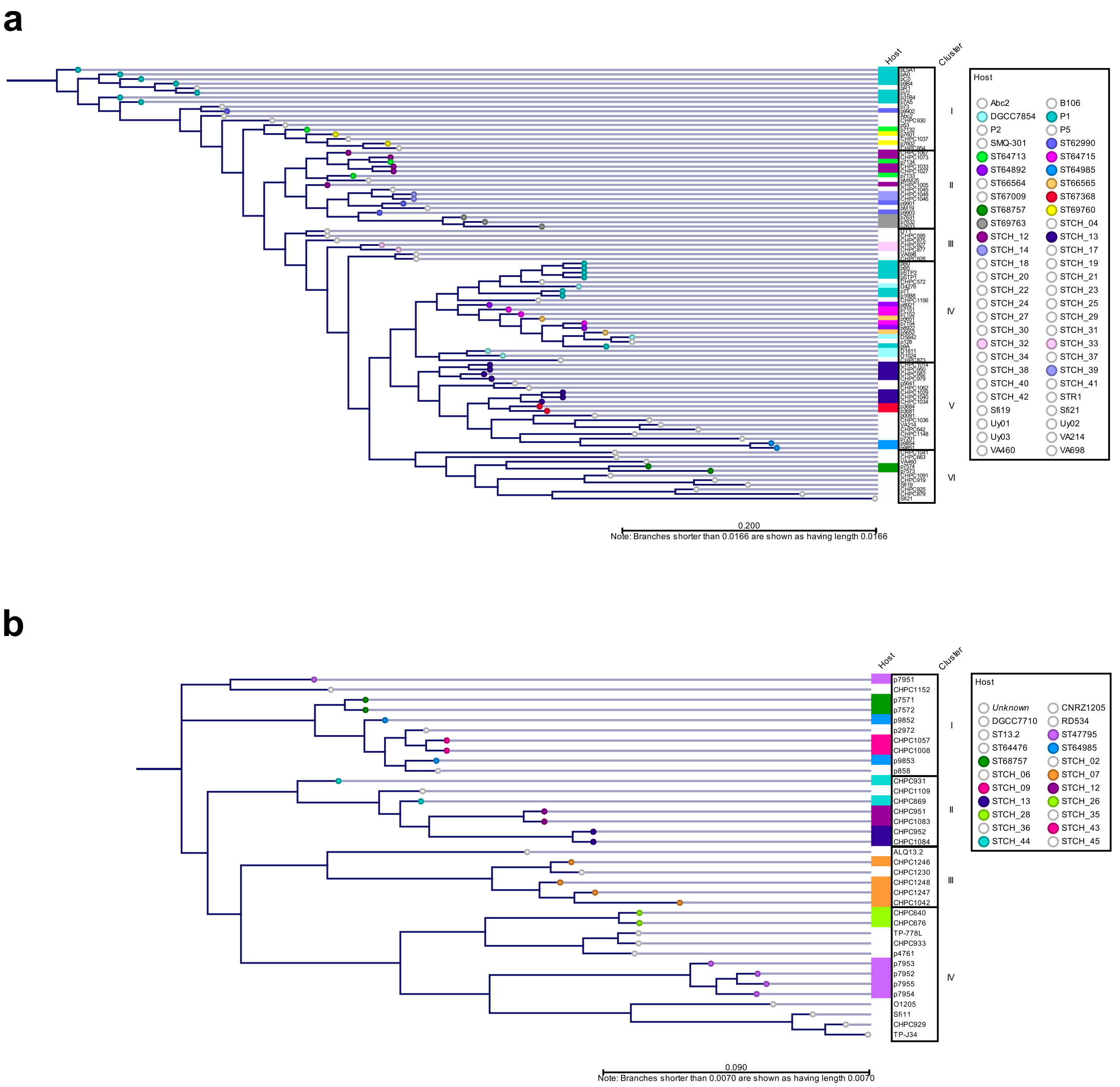
a
Host
Cluster
I
II
III
IV
V
VI
Host
Abc2
B106
DGCC7854
P1
P2
P5
SMQ-301
ST62990
ST64713
ST64715
ST64892
ST64985
ST66564
ST66565
ST67009
ST67368
ST68757
ST69760
ST69763
STCH_04
STCH_12
STCH_13
STCH_14
STCH_17
STCH_18
STCH_19
STCH_20
STCH_21
STCH_22
STCH_23
STCH_24
STCH_25
STCH_27
STCH_29
STCH_30
STCH_31
STCH_32
STCH_33
STCH_34
STCH_37
STCH_38
STCH_39
STCH_40
STCH_41
STCH_42
STR1
Sfi19
Sfi21
Uy01
Uy02
Uy03
VA214
VA460
VA698
0.200
Note: Branches shorter than 0.0166 are shown as having length 0.0166
b
Host
Cluster
p7951
CHPC1152
p7571
p7572
p9852
p2972
CHPC1057
CHPC1008
p9853
p858
CHPC931
CHPC1109
CHPC869
CHPC951
CHPC1083
CHPC952
CHPC1084
ALQ13.2
CHPC1246
CHPC1230
CHPC1248
CHPC1247
CHPC1042
CHPC640
CHPC676
TP-778L
CHPC933
p4761
p7953
p7952
p7955
p7954
O1205
Sfi11
CHPC929
TP-J34
I
II
III
IV
Host
Unknown
CNRZ1205
DGCC7710
RD534
ST13.2
ST47795
ST64476
ST64985
ST68757
STCH_02
STCH_06
STCH_07
STCH_09
STCH_12
STCH_13
STCH_26
STCH_28
STCH_35
STCH_36
STCH_43
STCH_44
STCH_45
0.090
Note: Branches shorter than 0.0070 are shown as having length 0.0070

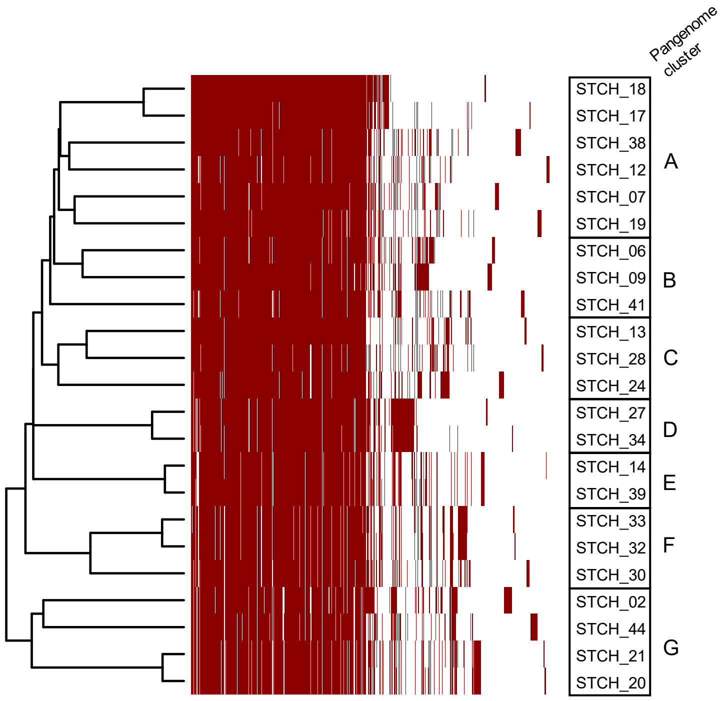
Pangenome cluster
STCH_18
STCH_17
STCH_38
STCH_12
STCH_07
STCH_19
A
STCH_06
STCH_09
STCH_41
B
STCH_13
STCH_28
STCH_24
C
STCH_27
STCH_34
D
STCH_14
STCH_39
E
STCH_33
STCH_32
STCH_30
F
STCH_02
STCH_44
STCH_21
STCH_20
G
